# Transcriptomic Analysis of the Regulatory Mechanism of Tea Polyphenol Biosynthesis in *Chionanthus retusus* and Functional Characterization of *CrHSP70-14* in Terms of Its Effect on Tea Polyphenols

**DOI:** 10.3390/metabo16010026

**Published:** 2025-12-25

**Authors:** Liyang Guo, Yuzhu Wu, Jihong Li, Haiyan Wang, Muge Niu, Mengmeng Wang, Shicong Zhao, Wenjing Song, Jiaxun Liu, Jingyu Wang, Jinnan Wang

**Affiliations:** 1 College of Forestry, Shandong Agricultural University, Tai’an 271018, China; 2023120308@sdau.edu.cn (L.G.); 2023120347@sdau.edu.cn (Y.W.); jhli@sdau.edu.cn (J.L.); 2023110230@sdau.edu.cn (M.W.); 2024110234@sdau.edu.cn (S.Z.); 2024120373@sdau.edu.cn (W.S.); 2025120398@sdau.edu.cn (J.L.); 2025120413@sdau.edu.cn (J.W.); 2 State Forestry and Grassland Administration Key Laboratory of Silviculture in Downstream Areas of the Yellow River, Tai’an 271018, China; 3 Heze Forestry Technical Service Center, Heze 274099, China; hzlylyz@hz.shandong.cn; 4 College of Forestry and Grassland, Nanjing Forestry University, Nanjing 210037, China; 2240100021@njfu.edu.cn

**Keywords:** *Chionanthus retusus*, tea polyphenol, regulatory network, *CrHSP70-14*

## Abstract

**Background:** *Chionanthus retusus Lindl. et Paxt.*, a deciduous tree of the genus Chionanthus (Oleaceae), represents a significant native species and a widely cultivated ornamental. Its tender leaves can be processed into tea, traditionally consumed in southern China under the common name “Nuomi Cha”. **Methods:** Our team quantified the tea polyphenol content across 150 individual trees of *C. retusus* and selected three low-polyphenol (ZB_D_14, AQ_2, AQ_1) and three high-polyphenol (SX_3, SXG_D_8, TS_D_13) lines for transcriptome sequencing of their young leaves. The resulting data were analyzed to screen for candidate genes. Subsequently, transgenic plants were constructed, and their tea polyphenol content was determined. **Results:** A significant difference in tea polyphenol content was confirmed between the high- and low-polyphenol lines. Weighted Gene Co-expression Network Analysis (WGCNA) pinpointed a key module strongly associated with tea polyphenol synthesis, encompassing 432 DEGs, which were predominantly enriched in pathways like phenylpropanoid biosynthesis. A comparative transcriptomic analysis further yielded 84 DEGs (40 up- and 44 down-regulated). Enrichment analysis showed these were primarily involved in flavonoid and phenylpropanoid biosynthesis pathways. Expression profiling of genes in the tea polyphenol biosynthetic pathway indicated that several key genes (e.g., *4CL*, *CHS*, *DFR*) were highly expressed in the high-content lines. A gene interaction network related to this synthesis identified 20 hub genes (e.g., *CrHSP70-14*, *CrMYB44*, *CrbHLH92*). Functional validation of four hubs (*CrMYB44*, *CrHSP70-14*, *CrCDC6B*, *CrRAE1*) via tobacco transient transformation assays demonstrated that all four significantly elevated tea polyphenol levels, with *CrHSP70-14* overexpression yielding the highest content. Furthermore, stable *CrHSP70-14* overexpression transgenic tobacco lines were generated, exhibiting significantly higher leaf tea polyphenol content versus controls. **Conclusions:** This study identifies multiple regulatory genes involved in *C. retusus* tea polyphenol biosynthesis, provides initial mechanistic insights, and establishes a molecular foundation for breeding specialized tea cultivars of this species.

## 1. Introduction

Tea, one of the world’s three major beverages, is consumed by approximately two-thirds of the global population [1]. Its widespread popularity is largely due to its diverse health benefits and unique flavor profile, which originate mainly from its rich abundance of secondary metabolites, including tea polyphenols (TPs), purine alkaloids, amino acids, and volatile compounds [2]. TPs, one of the primary secondary metabolites in tea, comprise six major compound classes: flavanones, anthocyanins, flavonols, flavan-3-ols (leucoanthocyanidins), phenolic acids, and depsides [3]. Among these, flavan-3-ols (mainly catechins) are the most predominant, accounting for 60–80% of total TPs, followed by flavonoids, while other phenolic classes are present in relatively minor amounts [3]. TPs exert multiple beneficial effects on human health. These compounds can mitigate cancer risk [4], ameliorate metabolic disorders [5], alleviate insulin resistance, activate antioxidant defenses, and promote wound healing in diabetes [6]. Concurrently, TPs have been shown to reduce myocardial fibrosis, improve cardiac function [7], and confer protective effects against stroke [8].

The biosynthesis of TPs originates from the phenylpropanoid and flavonoid pathways. Here, phenylalanine is sequentially catalyzed by *PAL*, *C4H*, and *4CL* to produce coumaroyl-CoA [9]. Leucoanthocyanidin is subsequently formed through various biological steps and converted to non-ester-type catechins via the leucoanthocyanidin reductase (*LAR*) pathway [10]. Esterification requires gallic acid activation to form galloylglucose by *UGGT*, followed by galloyl transfer to catechins, catalyzed by *ECGT*, to generate ester-type catechins [11]. Tea polyphenol (TP) biosynthesis is coordinately regulated by multiple factors, including environmental cues, plant hormones, transcription factors (TFs), and structural genes, with transcriptional regulation being pivotal [12,13]. Studies indicate that TP synthesis is governed by a hierarchical regulatory network involving various structural genes and TFs, such as *MYB*, *bHLH*, and *WD40* proteins [14]. In *Camellia sinensis*, the *R2R3-MYB* subfamily member *CsMYB34* specifically activates the acyltransferase gene *CsSCPL4*, thereby promoting TP synthesis [15]. The *CsbHLH89* protein interacts with *MYB* TFs to form complexes that co-activate key genes in the phenylpropanoid and flavonoid pathways, enhancing TP accumulation [16]. *CsWRKY12* directly binds to the promoters of the ester-type catechin biosynthesis genes *CsSCPL4* and *CsSCPL5*, activating their expression to drive the galloylation reactions producing *EGCG* and *ECG* [17]. Meanwhile, SBP/MADS-class TFs inhibit competing pathway genes (e.g., *CHS*, *F3H*, *ANS*), reducing flux into anthocyanin and proanthocyanidin branches and redirecting carbon toward catechin synthesis [18]. Furthermore, silencing *CsUGT84A* significantly reduces ester-type catechins (*EGCG*, *ECG*) and accumulates non-ester-type catechins (*EGC*, *EC*), confirming its role as a key rate-limiting enzyme in the esterification reaction [19].

*Chionanthus retusus Lindl. & Paxt.* is a deciduous tree of the genus *Chionanthus* (Oleaceae), classified as a national second-class protected plant in China and natively distributed across China, Korea, and Japan [20]. It exhibits remarkable ecological adaptability, possessing concurrent tolerance to drought, salinity, and waterlogging [21], making it a preferred species for reforesting barren land and urban greening. Its dense, snow-like spring inflorescences confer high ornamental value. The young leaves and flowers can be processed into a traditional herbal tea, locally known as “Nuomi Cha” [22]. The young leaves are rich in flavonoids (e.g., quercetin, kaempferol) and polyphenolic compounds, demonstrating significant antioxidant, anti-inflammatory, and neuroprotective activities, with potential intervention effects against chronic inflammation and neurodegenerative diseases [23]. However, research on TPs in *C. retusus* remains unreported. Therefore, this study measured TP content across different varieties, screening three high- and three low-content varieties for subsequent analysis of their young leaves. Transcriptome analysis was employed to identify differentially expressed genes (DEGs) and key regulatory genes controlling TP biosynthesis, providing a preliminary elucidation of the biosynthetic mechanism. To gain deeper insight, we generated transgenic tobacco plants overexpressing the key candidate gene *CrHSP70-14* involved in TP biosynthesis and analyzed its functional role in this process.

## 2. Materials and Methods

### 2.1. Plant Materials

Young leaves (the 4th to 8th leaves from the apical bud) were collected from the middle branches of 150 individual Chionanthus retusus plants in May 2023. All plant materials were derived from two-year-old grafted branches grown at the Shandong Agricultural University experimental field (36°10′ N, 117°9′ E). For each plant, approximately 5–10 g of leaf tissue was sampled for the determination of TP content. Based on the determination results, young leaves were sampled from three low-TP content lines (ZB_D_14, AQ_2, AQ_1) and three high-TP content lines (SX_3, SXG_D_8, TS_D_13) cultivated in the experimental field of Shandong Agricultural University. For each line, three biological replicates were sampled. All collected samples were immediately flash-frozen in liquid nitrogen and stored at −80 °C until subsequent RNA extraction for transcriptome sequencing.

### 2.2. Tea Polyphenol Extraction and Content Measurement

TP content was determined according to the Chinese National Standard GB/T8313-2008, using the ferrous tartrate colorimetric method. Precisely 1.00 g of leaf tissue from each of the six superior *Chionanthus retusus* accessions was weighed (three replicates per accession) and steeped in 45 mL of boiling water for 45 min to extract TPs. The resulting extracts were centrifuged at 3500 rpm for 10 min, and the supernatant was brought to a final volume of 50 mL. A 1 mL aliquot of the test solution was pipetted into a 25 mL volumetric flask, followed by the sequential addition of 5 mL deionized water and 5 mL ferrous tartrate solution, with thorough mixing after each addition. The mixture was diluted to the mark with phosphate buffer (pH 7.5). Absorbance was measured at 540 nm using a microplate reader.

### 2.3. Transcriptome Sequencing and Data Analysis

Young leaf samples from the low-TP (ZB_D_14, AQ_2, AQ_1) and high-TP (SX_3, SXG_D_8, TS_D_13) lines were selected and sent to Novogene Technology Co., Ltd. (Novogene Co., Ltd., Beijing, China) for transcriptome analysis. RNA quality was assessed using a Bioanalyzer (Agilent 2100, Santa Clara, CA, USA), and cDNA libraries were constructed. Raw sequencing reads underwent quality control using fastp. Clean reads were aligned to the *C. retusus* genome (sequenced in-house) using HISAT2. Transcript assembly was performed with StringTie v2.2.0, which employs a network flow algorithm and allows for de novo assembly; novel transcripts were annotated using Pfam, Superfamily, GO, and KEGG databases. Gene expression levels were quantified as FPKM (Fragments Per Kilobase of transcript per Million mapped reads). Principal Component Analysis (PCA) was performed on the FPKM values from all samples using linear algebra calculations. Differentially expressed genes (DEGs) were identified using the DESeq2 R package 1.20.0. Genes with raw counts < 10 across all samples were first filtered out. DEG screening was then performed using thresholds of an adjusted *p*-value (FDR an absolute log2 fold change ≥ 1.0. Enrichment analyses for DEGs were conducted using the GO and KEGG databases. A protein–protein interaction network) ≤ 0.05 and for the DEGs was analyzed using the STRING database and visualized in Cytoscape (v3.5.1). A gene co-expression network was constructed using the Weighted Gene Co-expression Network Analysis (WGCNA) algorithm.

### 2.4. Validation of Expression Patterns by Quantitative Real-Time PCR (qRT-PCR)

Fifteen genes associated with TP biosynthesis were selected for validation by qRT-PCR. Primer sequences were designed using Primer Premier 5.0. qRT-PCR reactions were performed using the SYBR Green Premix Pro Taq HS qPCR Kit AG96 on a CFX-2 Real-Time PCR Detection System (Bio-Rad, Hercules, CA, USA) integrated with a PCR instrument (biometra GmbH, analytik jena, Jena, Germany). The ubiquitin carrier protein 2 (*UBC2*) gene of *C. retusus* served as the internal reference. Relative gene expression levels were calculated using the 2^(−ΔΔCt) method. All primers are listed in Table S1.

### 2.5. Functional Validation of Tea Polyphenol-Related Genes from C. retusus in Tobacco

The coding sequences of four genes (*CrMYB44*, *CrHSP70-14*, *CrCDC6B*, and *CrRAE1*) were amplified from high-TP *C. retusus* lines using gene-specific primers and cloned into the pBI121 expression vector via homologous recombination. Recombinant vectors were sequenced for verification. Agrobacterium tumefaciens GV3101 harboring the recombinant vectors was used for transient transformation of tobacco leaves; TP content was measured three days post-infiltration. For stable transformation, the *CrHSP70-14* CDS was amplified and similarly cloned into pBI121, with the recombinant plasmid confirmed by sequencing. A. tumefaciens GV3101 carrying the vector was used for stable tobacco transformation via the leaf disc method [24]. Putative transgenic plants were cultured to seedling stage, and RNA was extracted for qRT-PCR to identify positive lines. TP content was ultimately determined in mature positive transgenic plants. All primers are listed in Table S1.

## 3. Results

### 3.1. Determination of Tea Polyphenol Content in High- and Low-TP Content Lines

**Figure 1:**
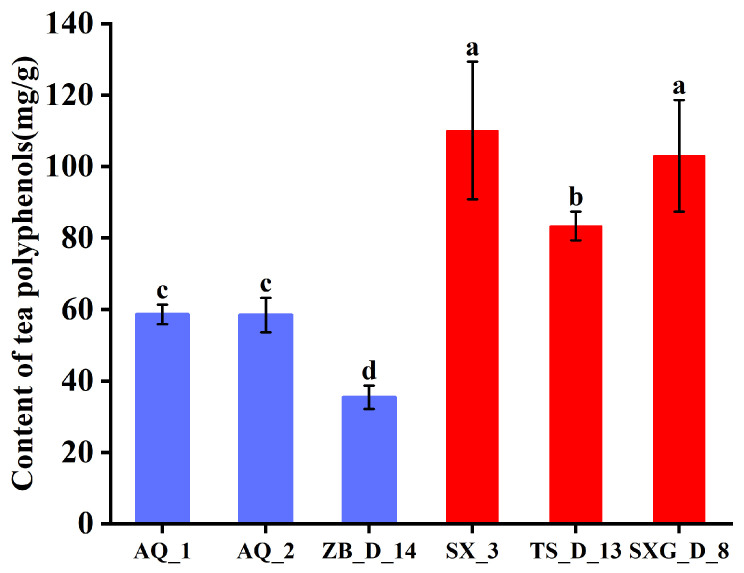
Tea polyphenol (TP) content in germplasms with high and low TP levels. Data are presented as mean ± standard deviation (*n* = 6). Significant differences among the lines (*p* < 0.05) are denoted by different lowercase letters (a–d), according to Duncan’s test.

Our preliminary study quantified the TP content in young leaves of 150 *Chionanthus retusus* individuals (unpublished data), revealing substantial variation among them. The TP content in lines AQ_1, AQ_2, and ZB_D_14 was significantly lower than that in lines SX_3, TS_D_13, and SXG_D_8 (Figure 1). The average TP content was 50.82 mg/g in the low-content lines and 98.81 mg/g in the high-content lines. The mean content in the high-content lines was approximately 90% greater than that in the low-content lines, indicating a pronounced disparity among the six selected lines.

### 3.2. Lines Transcriptome Sequencing and Differential Expression Gene Analysis

**Figure 2:**
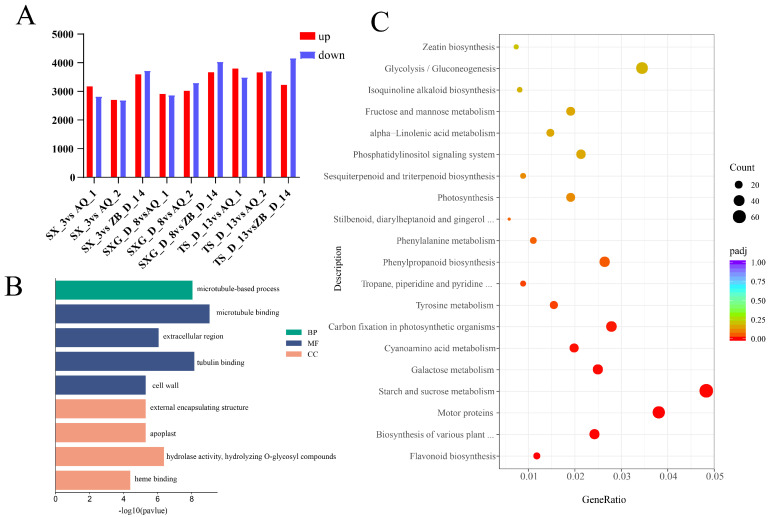
Statistical and functional enrichment analysis of differentially expressed genes (DEGs). (**A**) Number of up-regulated and down-regulated DEGs. (**B**) KEGG enrichment analysis of DEGs. (**C**) GO enrichment analysis of DEGs.

To investigate the molecular mechanism of TP biosynthesis in *C. retusus*, transcriptome sequencing was performed on young leaves from the six lines, yielding a total of 855,953,122 clean reads (Table S2). The Q30 percentage and GC content were 94.66% and 43.83%, respectively (Table S2). Principal component analysis (PCA) revealed that the first and second principal components (PC1 and PC2) were the major axes separating the samples. PC1 accounted for 23.92% of the total variance, while PC2 explained 18.80%, together contributing 42.72% of the cumulative variance and effectively capturing the major variation among samples. All biological replicates (three per line) clustered tightly within their respective line groups in the PCA plot. Furthermore, the transcriptome correlation coefficient between any two replicates exceeded 0.984 (Figure S1), demonstrating high within-group reproducibility and technical consistency of the experiment. Three low-TP lines (ZB_D_14, AQ_1, AQ_2) were each compared with three high-TP lines (SX_3, SXG_D_8, TS_D_13), revealing a substantial number of differentially expressed genes (DEGs) ranging from 5395 to 7705 across all pairwise comparisons. The comparison between SXG_D_8 and ZB_D_14 yielded the highest total number of DEGs (7705), while SX_3 vs. AQ_1 showed the greatest number of up-regulated genes (3174). In contrast, the comparison between TS_D_13 and ZB_D_14 contained the most down-regulated genes (4160) (Figure 2A). Existing studies have demonstrated that the biosynthesis of tea polyphenols primarily occurs through the flavonoid biosynthesis and phenylpropanoid biosynthesis pathways [25]. Gene Ontology (GO) enrichment analysis indicated that the DEGs were predominantly enriched in terms such as “cell wall and external encapsulating structure,” “xyloglucan xyloglucosyl transferase activity,” and “glycosyl bond hydrolase activity” (Figure 2B). Kyoto Encyclopedia of Genes and Genomes (KEGG) pathway analysis showed that among the top 20 enriched pathways, several were associated with TP synthesis, including flavonoid biosynthesis, phenylalanine metabolism, and phenylpropanoid biosynthesis (Figure 2C). In our research, differentially expressed genes were also predominantly enriched in these same pathways—flavonoid biosynthesis and phenylpropanoid biosynthesis. This alignment with established knowledge further indicates that tea polyphenol biosynthesis in Chionanthus retusus is largely derived from the flavonoid and phenylpropanoid biosynthesis pathways.

### 3.3. Co-Expression Network Analysis of Tea Polyphenols in High- and Low-Content Lines

**Figure 3:**
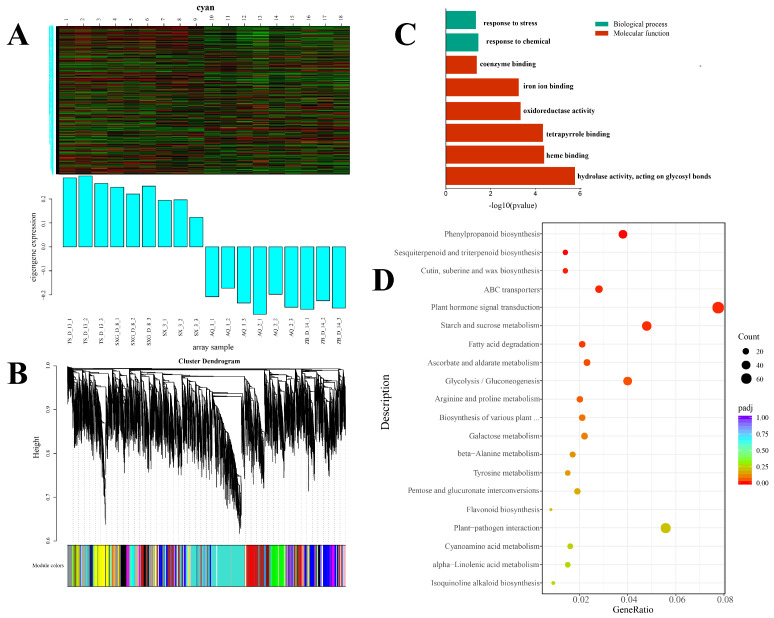
Weighted gene co-expression network analysis (WGCNA). (**A**) Expression patterns of genes within the cyan module. (**B**) Hierarchical clustering dendrogram of gene co-expression modules. (**C**) GO enrichment analysis for genes in the cyan module. (**D**) KEGG pathway enrichment analysis for genes in the cyan module.

To uncover potential associations between gene expression patterns and phenotypic traits, we employed Weighted Gene Co-expression Network Analysis (WGCNA) to construct a gene co-expression network and identify key functional modules and hub genes. WGCNA clustered the DEGs into 28 distinct modules (Figure 3B). The ‘Cyan’ module, comprising 486 genes and demonstrating consistently high expression across the three high-TP lines (SX_3, SXG_D_8, and TS_D_13), was selected for further analysis (Figure 3A). GO enrichment analysis of these 486 genes revealed significant enrichment in functional categories including hydrolase activity, coenzyme binding, and iron ion binding (Figure 3C). KEGG pathway analysis further indicated that these DEGs were predominantly enriched in pathways related to TP synthesis, including flavonoid biosynthesis, sesquiterpenoid and triterpenoid biosynthesis, and phenylpropanoid biosynthesis (Figure 3D). Further analysis of this module identified key genes within the tea polyphenol biosynthetic pathway, such as *PAL* and *CHS*. Additionally, the module contained genes associated with secondary metabolism, including members of the *MYB* transcription factor family.

### 3.4. Analysis of Key Genes in the Tea Polyphenol Biosynthetic Pathway

**Figure 4:**
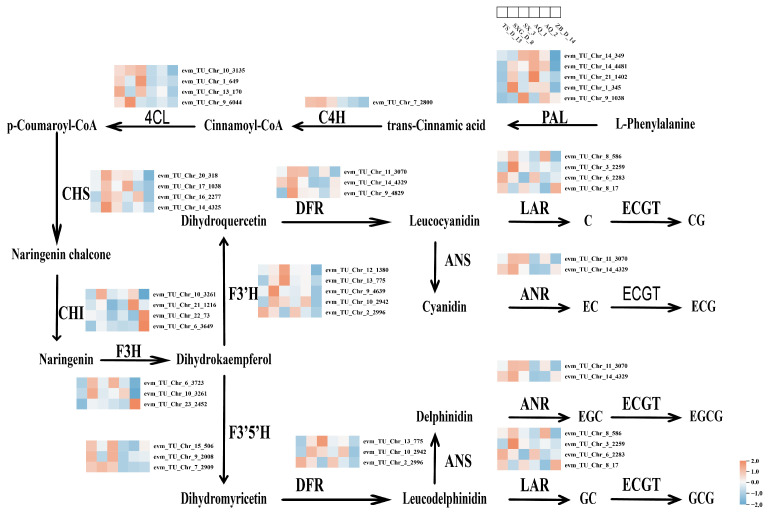
Proposed biosynthetic pathway for tea polyphenols in Chionanthus retusus. Expression trends of key structural genes are indicated adjacent to their corresponding enzyme symbols. Abbreviations: *PAL*, phenylalanine ammonia-lyase; *C4H*, cinnamate-4-hydroxylase; *4CL*, 4-coumarate-CoA ligase; *CHS*, chalcone synthase; *CHI*, chalcone isomerase; *F3H*, flavanone 3-hydroxylase; *F3′H*, flavonoid 3′-hydroxylase; *F3′5′H*, flavonoid 3′,5′-hydroxylase; *DFR*, dihydroflavonol 4-reductase; *ANS*, anthocyanidin synthase; *LAR*, leucoanthocyanidin reductase; *ANR*, anthocyanidin reductase; *ECGT*, epicatechin:O-galloyltransferase; C, catechin; *CG*, catechin gallate; *EC*, epicatechin; *ECG*, epicatechin gallate; *EGC*, epigallocatechin; *EGCG*, epigallocatechin gallate; *GC*, gallocatechin; *GCG*, gallocatechin gallate.

Based on transcriptomic data, the TP biosynthetic pathway in *C. retusus* was mapped. The results revealed a consistent expression pattern for genes involved in the pathway from L-phenylalanine to catechin gallates (e.g., *PAL*, *4CL*, *CHS*, *F3′5′H*, *DFR*, *ANR*, and *LAR*) (Figure 4). The expression levels of key rate-limiting enzyme genes (*PAL*, *CHS*, *ANR*, and *LAR*) [26,27,28,29] were significantly higher in the high-TP lines (SX_3, SXG_D_8, TS_D_13) than in the low-TP lines (ZB_D_14, AQ_2, AQ_1). These enzymes are established as crucial regulators collectively controlling TP biosynthesis: *PAL* is the initial key enzyme in the phenylpropanoid pathway, providing core precursors; *CHS* is the first key enzyme in the flavonoid pathway, determining flavonoid synthesis rate and metabolic flux; *LAR* and *ANR* catalyze the production of non-gallated catechins downstream, regulating the final TP composition. The differential expression of these key enzymes explains the higher TP content in the high-content lines and underscores the critical roles of *PAL*, *CHS*, and *LAR* in this process.

### 3.5. Comparative Transcriptome Analysis of Differentially Expressed Genes

**Figure 5:**
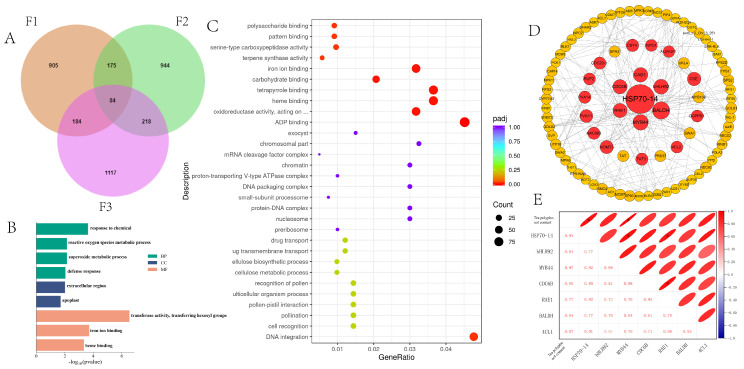
Regulatory mechanisms of tea polyphenol synthesis in high- and low-content genotypes. (**A**) Venn diagram showing common differentially expressed genes (DEGs) among comparison groups F1, F2, and F3. (**B**) Top 20 significantly enriched GO terms for the 84 common DEGs. (**C**) Top 20 significantly enriched KEGG pathways for the 84 common DEGs. (**D**) Protein–protein interaction (PPI) network of tea polyphenol-related genes. (**E**) Heatmap showing correlations between expression levels of key candidate genes and tea polyphenol content. The asterisk (∗) indicates significant difference (*p* < 0.05).

To further investigate the molecular basis of TP synthesis, we conducted an in-depth comparative transcriptome analysis. Gene sets were defined as follows: F1 represents the intersection of DEGs from comparisons of SX_3 versus ZB_D_14, AQ_2, and AQ_1; F2 from SXG_D_8 versus AQ_1, AQ_2, and ZB_D_14; and F3 from TS_D_13 versus AQ_1, AQ_2, and ZB_D_14. Screening identified 84 common DEGs shared across the F1, F2, and F3 gene sets (Figure 5A). KEGG enrichment analysis of these 84 DEGs showed significant enrichment in starch and sucrose metabolism, plant secondary metabolite biosynthesis, flavonoid biosynthesis, and phenylpropanoid biosynthesis (Figure 5B). GO enrichment analysis indicated primary association with heme binding, oxidoreductase activity, and hexosyl group transferase activity (Figure 5C). A TP interaction regulatory network was constructed using Cytoscape, from which the top 16 genes with the highest connectivity scores were selected as candidate hub genes (Figure 5D). These include 3 transcription factors (*CrMYB44*, *CrbHLH92*, *CrNAC083*), 11 metabolic enzymes (*CrCAD1*, *CrTKT3*, *CrNPC4*, *Cr4CL2*, *CrBALDH*, *CrALDH3F1*, *CrCSE*, *CrGGPPS1*, *CrHOMT3*, *CrRUP2*, *CrRAE1*), 5 structural proteins (*CrCDC20-1*, *CrCDC6B*, *CrCSY4*, *CrPVA11*, *CrPVA14*), and 1 molecular chaperone (*CrHSP70-14*). Correlation analysis revealed that TP content was significantly positively correlated with the expression levels of *CrHSP70-14* and *CrMYB44*. The expression pattern of *CrHSP70-14* showed positive correlation with both *CrMYB44* and *CrRAE1*, and *CrRAE1* expression was positively correlated with *CrCDC6B*, indicating strong mutual positive correlations among these genes (Figure 5E).

### 3.6. Validation of Differentially Expressed Gene Expression Patterns

**Figure 6:**
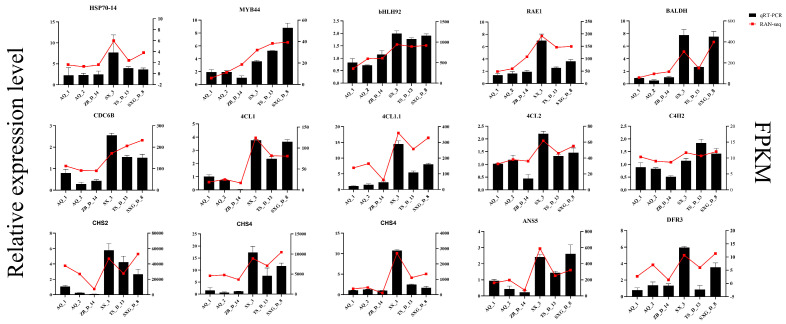
Validation of expression patterns for 15 differentially expressed genes. Bar charts compare relative expression levels from qRT-PCR with FPKM values from RNA-Seq for selected genes. *UBC2* served as the internal reference gene, and expression level of each target gene in AQ_1 was set to 1. Data represent mean ± standard deviation (*n* = 15).

To validate the accuracy and reproducibility of the transcriptome data, 15 genes involved in TP biosynthesis regulation, including *CrHSP70-14*, *CrBALDH*, *CrbHLH92*, *CrMYB44*, *CrRAE1*, *CrCDC6B*, and Cr4CL1, were selected for qRT-PCR analysis (Figure 6). The results demonstrated strong concordance between qRT-PCR measurements and the corresponding FPKM values. Notably, *CrHSP70-14* exhibited significantly upregulated expression in the high-TP lines. Most genes analyzed showed consistent expression patterns between the two methodologies, confirming the reliability of the RNA-Seq data.

### 3.7. Effects of Overexpressing CrHSP70-14, CrMYB44, CrCDC6B, and CrRAE1 on Tea Polyphenol Content in Tobacco

**Figure 7:**
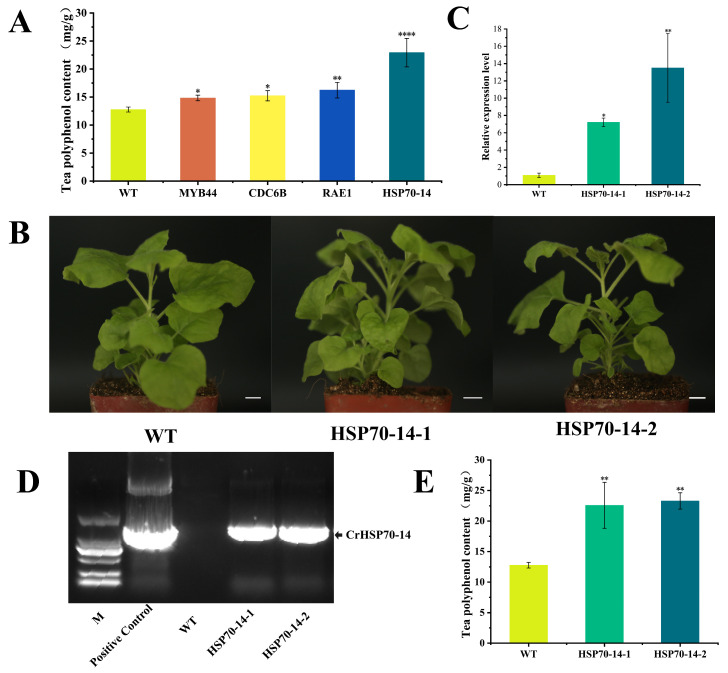
Phenotypic and molecular characterization of *CrHSP70-14* transgenic tobacco lines. (**A**) Tea polyphenol content in wild-type and tobacco leaves transiently expressing the four candidate genes. (**B**) Phenotype comparison of two independent *CrHSP70-14* overexpression lines with wild-type control. (**C**) Relative expression levels of *CrHSP70-14* in transgenic lines. (**D**) PCR validation of the *CrHSP70-14* transgene in putative transgenic plants. M: Marker 2000 molecular weight marker; Positive Control: pBI121 overexpression vector harboring the *CrHSP70-14* gene. (**E**) Tea polyphenol content in stable *CrHSP70-14* overexpression lines versus wild-type control. Asterisks indicate significant differences compared to WT (*t*-test): * *p* < 0.05, ** *p* < 0.01, **** *p* < 0.0001.

To further elucidate the molecular mechanism of TP biosynthesis, we investigated the function of four hub genes (*CrMYB44*, *CrHSP70-14*, *CrCDC6B*, and *CrRAE1*) using tobacco transient transformation assays. The results demonstrated that TP content in tobacco leaves transiently expressing each of the four hub genes was significantly higher than in the control (Figure 7A). Among them, transient overexpression of *CrHSP70-14* resulted in the highest TP accumulation (22.9 mg/g), followed by *CrRAE1*. The control group showed the lowest content (approximately 12.76 mg/g), indicating a significant difference compared to all four gene transformations. These results confirm that *CrMYB44*, *CrCDC6B*, *CrRAE1*, and *CrHSP70-14* all positively regulate TP biosynthesis. To further validate the role of the candidate gene, we generated stable *CrHSP70-14* overexpression transgenic tobacco lines (Figure 7B). PCR and qPCR analyses confirmed the successful generation of two independent transgenic lines, exhibiting 4.4-fold and 9.7-fold upregulation of *CrHSP70-14* expression, respectively (Figure 7C,D). TP content was significantly higher in both transgenic lines compared to the control. The *CrHSP70-14*-1 line accumulated 22.58 mg/g (1.76-fold increase over control), while the *CrHSP70-14*-2 line reached 23.28 mg/g (1.82-fold increase) (Figure 7E). This result conclusively demonstrates that *CrHSP70-14* significantly enhances TP biosynthesis.

## 4. Discussion

As a traditional native Chinese tree species, *Chionanthus retusus* shows considerable potential for development as a novel, alternative tea resource, with its leaves serving as the primary material. Tea polyphenols (TPs) represent a key indicator for evaluating the organoleptic quality (color, aroma, taste) and health benefits of tea plants, making the elucidation of their biosynthetic pathway crucial for the genetic improvement and industrial utilization of tea-oriented *C. retusus* varieties. Our analysis revealed significant variation in TP content among different lines, with the ZB_D_14 line exhibiting the lowest content (35.41 mg/g) and the SX_3 line the highest (110.09 mg/g) (Figure 1). Notably, the TP content in SX_3 (110.09 mg/g) is comparable to previously reported levels in green tea (126.59 ± 2.09 mg/g) [30], indicating that young leaves of high-polyphenol *C. retusus* lines approach the polyphenol levels of commercial green tea and confirming the species’ potential as a tea resource. Subsequent transcriptome sequencing of young leaves from three low-TP (ZB_D_14, AQ_2, AQ_1) and three high-TP (SX_3, SXG_D_8, TS_D_13) lines identified 58,563 differentially expressed genes (DEGs) potentially involved in TP biosynthesis in *C. retusus*.

WGCNA clusters genes with similar expression patterns [31] and constructs co-expression networks to identify modules of co-expressed genes. We selected the Cyan module, comprising 486 genes (Figure 3A), which exhibited significantly low expression in low-TP lines and significantly high expression in high-TP lines. Genes within this module were significantly enriched in pathways related to TP biosynthesis, such as flavonoid and phenylpropanoid biosynthesis. To further identify key enzymes in the TP biosynthetic pathway of *C. retusus*, we mapped the pathway, revealing that high expression levels of *C4H* and *4CL* lead to the production of p-coumaroyl-CoA, providing abundant precursor metabolites for TP synthesis [32]. This likely constitutes a key reason for the high TP content in leaves of SX_3, SXG_D_8, and TS_D_13. Chalcone synthase (*CHS*) and chalcone isomerase (*CHI*) catalyze the conversion of p-coumaroyl-CoA to naringenin [33]. The high expression of *CHS* in high-TP lines facilitates substantial naringenin biosynthesis, supplying essential intermediates for downstream TP production. *F3H*, *F3′5′H*, *F3′H*, and *DFR* are crucial enzymes in the TP pathway, catalyzing the formation of leucodelphinidin and leucocyanidin [34,35]. The elevated expression of *DFR* in high-content lines promotes the biosynthesis of these leucoanthocyanidins. All differentially expressed *DFR* genes showed higher expression in high-content lines, indicating that *DFR* serves as the primary reductase in *C. retusus*, playing a pivotal role in TP biosynthesis by catalyzing leucodelphinidin and leucocyanidin formation. Leucoanthocyanidin reductase (*LAR*) utilizes leucoanthocyanidins to produce catechin and gallocatechin [36], while anthocyanidin reductase (*ANR*) uses anthocyanidins to yield epicatechin and epigallocatechin [37]. These non-gallated catechins subsequently serve as precursors for ester-type catechin formation via esterification catalyzed by *ECGT*.

TP biosynthesis is a complex physiological and biochemical process regulated by multiple pathways involving environmental and genetic factors [38,39]. Elucidating this mechanism is crucial for understanding TP synthesis regulation and provides a theoretical foundation for breeding tea-oriented *C. retusus* with improved quality. To investigate potential regulatory genes, we constructed a gene co-expression network (Figure 5D) and selected the top 20 genes by connectivity as candidates. These included 3 transcription factors (*CrMYB44*, *CrbHLH92*, *CrNAC083*), 11 metabolic enzymes (*CrCAD1*, *CrTKT3*, *CrNPC4*, *Cr4CL2*, *CrBALDH*, *CrALDH3F1*, *CrCSE*, *CrGGPPS1*, *CrHOMT3*, *CrRUP2*, *CrRAE1*), 5 structural proteins (*CrCDC20-1*, *CrCDC6B*, *CrCSY4*, *CrPVA11*, *CrPVA14*), and 1 molecular chaperone (*CrHSP70-14*). Integrated correlation and expression pattern analyses suggested that *CrMYB44*, *CrbHLH92*, *CrNAC083*, *CrCDC6B*, *CrRAE1*, and *CrHSP70-14* play significant roles in TP synthesis. Numerous studies show that CsMYB4a can specifically bind to promoters of key catechin synthesis genes like *ANS*, *ANR*, and *LAR*, directly activating their transcription and enhancing TP content [40]. Furthermore, research across plant species indicates that *MYB* transcription factors participate conservatively in TP biosynthesis regulation [41,42], recognizing that cis-acting elements in target gene promoters coordinately activate anthocyanin and proanthocyanidin pathways, thereby promoting TP accumulation [43]. Here, the expression pattern of *CrMYB44* showed significant positive correlation with TP content. Subsequent functional validation via transient expression in tobacco significantly increased TP levels, confirming that *CrMYB44* promotes TP biosynthesis. The RAE family belongs to the F-BOX protein family, encoding nucleus-localized F-box proteins with important roles in plant stress resistance [44]. We found that *CrRAE1* was highly expressed in high-TP lines and its expression level correlated with TP biosynthesis, suggesting its potential involvement. This was confirmed by transient overexpression in tobacco, which increased TP content, indicating *CrRAE1* is a positive regulator. *HSP70* family genes are primarily known for roles in protein homeostasis and heat stress response [45,46], with no prior reports linking them to TP synthesis regulation. In our research, *CrHSP70-14* expression was 1.82-fold higher in high-content lines and showed significant positive correlation with TP content. Through both transient transformation and stable *CrHSP70-14* transgenic tobacco lines, we demonstrated that its overexpression significantly enhances TP levels, establishing *CrHSP70-14* as a positive regulator.

## 5. Conclusions

In summary, this study conducted transcriptome analysis on young leaves of *Chionanthus retusus* with low- and high-TP contents, revealing that the DEGs were predominantly enriched in phenylpropanoid and flavonoid biosynthetic pathways. Through WGCNA and a TP interaction regulatory network, we identified genes highly correlated with TP content, including transcription factors, metabolic enzymes, and molecular chaperones such as *CrMYB44*, *CrbHLH92*, *CrCDC6B*, *CrRAE1*, and *CrHSP70-14*. Agrobacterium-mediated transient transformation assays demonstrated that transient expression of *CrMYB44*, *CrCDC6B*, *CrHSP70-14*, and *CrRAE1* in tobacco leaves significantly enhanced TP accumulation. Stable *CrHSP70-14* overexpression transgenic tobacco lines exhibited significantly higher leaf TP content compared to wild-type controls. Collectively, this research provides novel insights into the TP biosynthetic mechanism in *C. retusus*, establishes a transcriptional regulatory network, and offers a preliminary elucidation of the molecular regulatory mechanism underlying TP biosynthesis in this species.

## Data Availability

The genomic information of the Chionanthus retusus mentioned in the article is available from the corresponding author upon reasonable request. This assembly used HiC and PacBio methods. The Hi-C and ONT data, as well was the assemblies have been deposited to China National GeneBank DataBase with Bioproject ID of CNP0008503.

## Supplementary Materials

The following supporting information can be downloaded at https://www.mdpi.com/article/10.3390/metabo16010026/s1, Figure S1: Principal component analysis (PCA) of transcriptome samples. The horizontal axis represents PC1, the vertical axis represents PC2, indicating scores of the first and second principal components, respectively; Table S1: Summary of RNA-Seq data filtering statistics; Table S2: List of primers used for qRT-PCR analysis.

## Abbreviations

The following abbreviations are used in this manuscript:

TP(s)	Tea Polyphenol(s)
DEG(s)	Differentially Expressed Gene(s)
WGCNA	Weighted Gene Co-expression Network Analysis
FPKM	Fragments Per Kilobase of transcript per Million mapped reads
PCA	Principal Component Analysis
GO	Gene Ontology
KEGG	Kyoto Encyclopedia of Genes and Genomes
qRT-PCR	Quantitative Real-Time Polymerase Chain Reaction
