# Supplementary material for: Transcriptomic Analysis of the Regulatory Mechanism of Tea Polyphenol Biosynthesis in Chionanthus retusus and Functional Characterization of CrHSP70-14 in Terms of Its Effect on Tea Polyphenols
Source: Metabolites. 2025 Dec 25;16(1):26. doi: 10.3390/metabo16010026 (PMC12843654; doi:10.3390/metabo16010026)
Supplement: Supplementary file 1 [file metabolites-16-00026-s001.zip › metabolites-4047152-supplementary.pdf]

Transcriptomic Analysis of the Regulatory Mechanism of Tea Polyphenol Biosynthesis in *Chionanthus retusus* and Functional Characterization of *CrHSP70-14* in Terms of Its Effect on Tea Polyphenols

Liyang Guo[1,2†], Yuzhu Wu[1,2†], Jihong Li[1,2], Haiyan Wang[3] , Muge Niu[4], Mengmeng Wang [1,2], Shicong Zhao[1,2], Wenjing Song [1,2], Jiaxun Liu[1,2], Jingyu Wang[1,2], Jinnan Wang[1,2]*

[1] College of Forestry, Shandong Agricultural University, Tai'an, Shandong Province 271018, China.

[2] State Forestry and Grassland Administration Key Laboratory of Silviculture in Downstream Areas of the Yellow River, Tai'an, Shandong Province 271018, China.

[3] Heze Forestry Technical Service Center, Heze Shandong Province 274099, China.

[4] College of Forestry and Grassland, Nanjing Forestry University, Nanjing Jiangsu Province 210037, China.

*Correspondence: Wjn@sdau.edu.cn

Appendix Figures

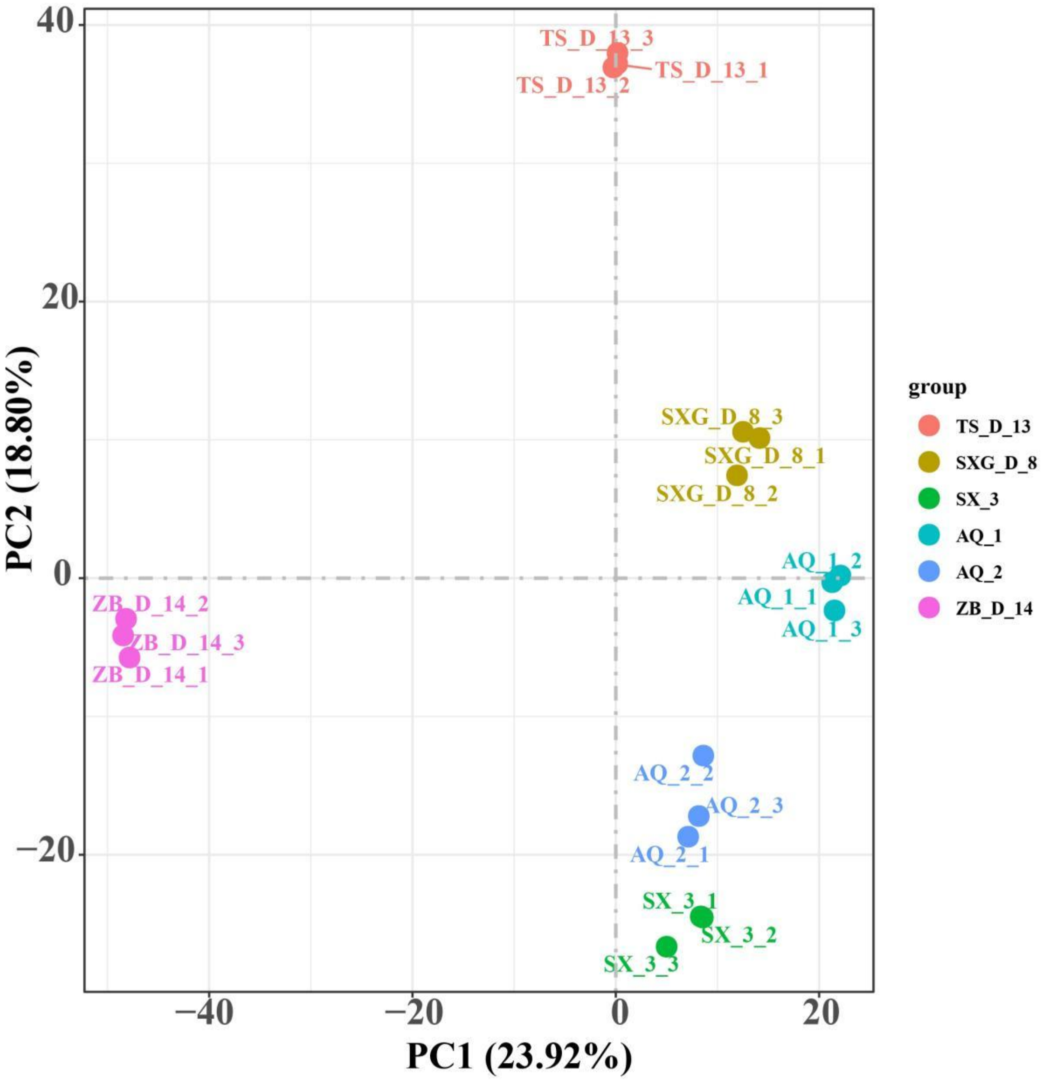

Figure S1. Principal component analysis (PCA) of transcriptome samples. Figure S1. Principal component analysis (PCA) of transcriptome samples. The horizontal axis represents PC1, the vertical axis represents PC2, indicating scores of the first and second principal components, respectively.

Appendix Tables

Table S1. Summary of RNA-Seq data filtering statistics.

Symbol by Swiss-Prot	Primer
HSP70-14-F	TCCAATGAGCACGAAGT
HSP70-14-R	GCGGCAACGAAGGT
CAD1-F	GGGTTGCTCCGAAAA
CAD1-R	TCCCTGACCTGCCTAT
bHLH92-F	GCTAACAAGGCGACAAG
bHLH92-R	CAAGAAACCAAGCCACTA
BALDH-F	TGTTCTGTTCCCGACTT
BALDH-R	CAATGCTGCTGTGCC
MYB44-F	AGGGTGACGCCAAGA
MYB44-R	CACTACGAACTCCGATGC
RAE1-F	GGGAAAGCAGCTAAACA
RAE1-R	TTCGTGGGATAGTCAGG
CDC6B -F	CCAACTCATCCGCAAT
CDC6B -R	TATGTATGTGGGTGTCCG
4CL1 -F	CGGCGAGTTATGGAGTA
4CL1 -R	GCCAACGGCGAAGTA
CHS2-F	TCAGAACCCACAATCACG
CHS2-R	GCCGACTACCAACTCACC
CHS4-F	CAATCACCGCTCGTC
CHS4-R	CCCAACTCAAATGCTG
4CL1.1-F	TGAAATAACGGTAACACGAT
4CL1.1-R	AAGCCGACCCAAAGG
C4H2-F	ATGTCCAAATCCTCCC
C4H2-R	CTCATAAGACACCCAACC
CHS4.1-F	TTTGTTGTTCTCCGCTAG
CHS4.1-R	GGCTCCGTCCTTCTGT
4CL2-F	CAGGGCAGTTCTTGG
4CL2-R	TCGGACATTTGTGAGC
DFR3-F	CCTCCGAGTGAAATGG
DFR3-R	AAGGAAGTTCGTGGCA
ANS5-F	AAGGGAGATAGTGAAGGAAGTA
ANS5-R	CAGACAGTGGCATAACAGC
UBC2-F	GTGGAGTGTGGAGGATAAGGGTG
UBC2-R	TGTTGACAAAACCGAGGAAGGA
MYB44-OE-F	ATGACGATGGTGCAAAAATCCG

MYB44-OE-R	CTACTTATGGAATTCCATGACCCAAAAG
CDC6B-OE-F	ATGCCGTCCATCGCCG
CDC6B-OE-R	TTATTGAAGACAACTCCGAAAGAAACGG
RAE1-OE-F	ATGGCTACGTTCGGCACTAC
RAE1-OE-R	TCACTTTCTACCACCTGTTCCAAT
HSP70-14-OE-F	ATGGAAACCCATACCATTCAACTCTCA
HSP70-14-OE-R	CTAGTAGTAAAACACAGAAAAGTTGAATGCCA

Table S2. List of primers used for qRT-PCR analysis.

sample	raw_reads	clean_reads	Q20	Q30	GC_pct
TS_D_13_1	41138672	40251176	98.08	94.45	43.93
TS_D_13_2	48508810	47270630	98.35	95.13	43.91
TS_D_13_3	47311748	45142978	98.31	95.05	43.95
SXG_D_8_1	50886624	49835974	98.07	94.44	43.85
SXG_D_8_2	43979626	43015012	98.05	94.4	43.69
SXG_D_8_3	48701448	47714234	98.19	94.75	43.77
SX_3_1	46656236	45646434	98.27	94.85	43.89
SX_3_2	53374410	52300782	97.96	94.11	43.95
SX_3_3	49444524	48102516	98.14	94.64	43.6
AQ_1_1	49325334	48462184	98.29	94.99	43.72
AQ_1_2	49575286	48477580	98.16	94.65	43.78
AQ_1_3	46807478	45626032	98.36	95.16	43.56
AQ_2_1	45313006	43779352	98.06	94.28	43.88
AQ_2_2	44918662	42843766	98.1	94.57	44.03
AQ_2_3	47799850	46507648	98.34	95.1	44.03
ZB_D_14_1	43980466	42961312	98.02	94.32	43.7
ZB_D_14_2	51292402	50188108	98.04	94.36	43.72
ZB_D_14_3	46938540	45625348	98.32	95.01	43.79
